# Comparison of calving and revenue-generating qualities in beef-sired male and female progeny from dairy cows

**DOI:** 10.3168/jdsc.2023-0395

**Published:** 2023-08-19

**Authors:** D.P. Berry, S. Ring

**Affiliations:** 1 Teagasc, Animal & Grassland Research and Innovation Centre, Moorepark, Fermoy P61 P302, Co. Cork, Ireland; 2 Irish Cattle Breeding Federation, Carrigrohane, Ballincollig, Co. Cork, P31 D452, Ireland

## Abstract

Although interest in beef-on-dairy breeding strategies is intensifying, little is actually known of the performance differences between beef-sired male and female progeny of dairy cows. The objective was therefore to use a large cross-sectional database of up to 1,389,670 animals to investigate if performance differences existed between male and female progeny generated from beef-on-dairy matings; the focus was on characteristics of interest to both the dairy producer (i.e., gestation length, calving performance, perinatal mortality, and calf sale value) and the beef producer (i.e., slaughter-related traits). While statistical differences existed between both sexes, the observed differences were not always biologically large, with some favoring females (e.g., calving traits and age at slaughter) and some favoring males (i.e., carcass weight). Beef-sired male calves had, on average, a 0.8 d longer gestation than their female counterparts; the sex difference in dairy-sired calves was, on average, 1.1 d, with the advantage to females. The odds of a difficult calving was 2.2 times greater for beef-sired male calves relative to beef-sired female calves; this translated to a difference in predicted probability of dystocia between the sexes of 1.8 percentage units. Male beef-sired calves sold at auctions <42 d of age were worth, on average, €32.40 more than beef-sired female calves. Focusing just on beef-sired progeny, relative to heifer carcasses (mean weight of 280.0 kg), the carcasses of steers (mean weight of 336.9 kg) and bulls (mean weight of 335.4) were 55.4 to 56.9 kg heavier. Based on a 15-point conformation scale, the carcasses of bulls were 1 unit superior to heifers, with the carcasses of the latter being 0.06 units better than steers. Heifers were slaughtered, on average, 79.1 d younger than steers although heifers were slaughtered, on average, 93.8 d older than bulls, the latter generally being finished on a more intensive diet relative to steers and heifers in Ireland. In conclusion, many benefits exist for beef-sired heifer calves in that they had, on average, shorter gestations with less expected assistance required at calving and, although their calf value was less and their carcasses were lighter than their male counterparts, they were slaughtered several months younger than steers.

## Highlights

• Beef-sired heifers had shorter gestations and fewer calving complications than males.
• Beef-sired heifer calves were less valuable than males.
• Heifers had lighter carcasses but were slaughtered younger than steers.

## Graphical Abstract

**Figure:**
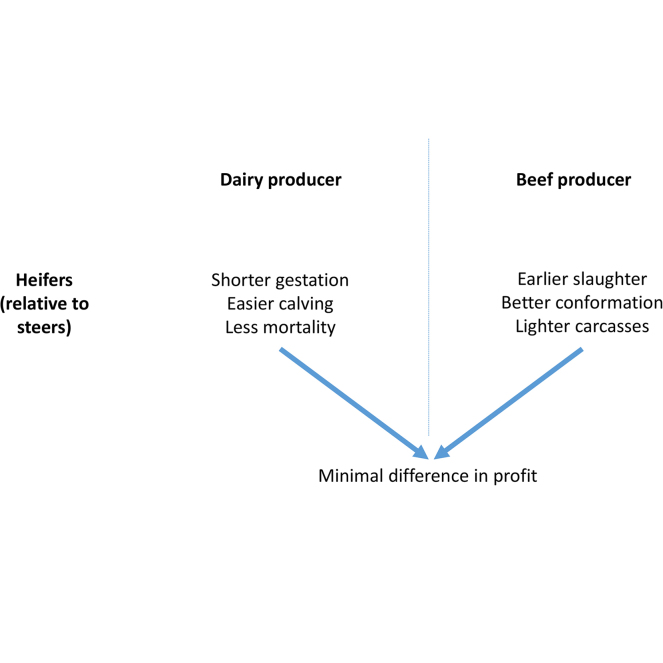
**Summary:** Interest is rapidly intensifying in the use of sex-sorted semen of dairy bulls; this sorting technology, however, could also be used to sort semen of beef bulls. Results from the cross-sectional analysis of >1.3 m records from animals born in dairy herds demonstrated that beef-sired heifer calves were born with a shorter gestation and required less calving assistance; the heifers were slightly less valuable when traded as calves, and although they had carcasses approximately 55 kg lighter than their male counterparts, they were slaughtered 79 days younger than their steer contemporaries.

Uptake in the mating of beef bulls to dairy females is growing (Van Doormaal, 2019; Berry and Ring, 2020b). The capacity to mate proportionally more females in a dairy herd to semen from beef sires is aided by numerous technological advances (Berry, 2021), not least the availability of X-chromosome sorted semen to generate proportionally more dairy-bred females. The same sexing technology, of course, could equally well be applied to semen from beef bulls to generate proportionally more males or females based on the desires and market opportunities of the dairy producer. To this end, it is important to establish the difference in performance between beef × dairy males and females with a particular focus on implications for dairy production systems (i.e., calving performance traits and calf price) and beef production systems (i.e., slaughter traits including carcass value).

While performance differences between male and female cattle have already been documented for a plethora of traits like gestation length (Norman et al., 2009), calving difficulty (Mee et al., 2011), perinatal mortality (Mee et al., 2008), and carcass-related traits (Kenny et al., 2020), most of these studies were based on either dairy-on-dairy matings only (Norman et al., 2009), did not describe exactly what matings were included (Johanson and Berger, 2003), or did not explicitly examine or test for an interaction between calf sex effects and whether the sire used was of dairy or beef origin (Mee et al., 2008, 2011; Kenny et al., 2020). Eriksson et al. (2020) did, however, specifically focus on performance metrics from beef-on-dairy matings, albeit most of the focus in that study was on comparing breeds of beef sires. Fouz et al. (2013) compared the gestation length and calving performance of male and female offspring from Holstein × Holstein, Holstein × Limousine, Holstein × Belgian Blue, and Holstein × Galician Blonde matings albeit did not differentiate the sex effects for the dairy-on-dairy versus beef-on-dairy matings. In general, male calves have a longer gestation (Norman et al., 2009; Fouz et al., 2013), are more prone to calving complications (Johanson and Berger, 2003; Mee et al., 2011; Fouz et al., 2013), and are more likely to die at or soon after birth (Johanson and Berger, 2003; Mee et al., 2008). Also, carcasses of male cattle are generally heavier than those of heifers (Kenny et al., 2020); this difference in carcass value is mirrored in the greater price paid for male calves (<42 d of age) from dairy herds relative to female calves, once breed differences are accounted for (Mc Hugh et al., 2010).

The objective of the present study was to use a large cross-sectional database of up to 1,389,670 animals to investigate if calving- and slaughter-related performance differences existed between male and female progeny from beef-on-dairy matings; progeny from dairy-on-dairy matings were also included for comparative purposes but especially to test for an interaction between mating type (i.e., beef × dairy vs. dairy × dairy) and sex.

All data used in the present study originated from the Irish Cattle Breeding Federation (www.icbf.com). Predicted transmitting ability values from the national multi-breed genetic evaluations in 2014 were available for direct and maternal calving difficulty, direct gestation length, and direct perinatal calf mortality, as well as the carcass traits of carcass weight, conformation, and fat score. All PTA are expressed relative to a common base population and thus are comparable across breeds. The EBV of each animal was calculated as the PTA of the sire plus half the PTA of the maternal grandsire.

Phenotypic data were available on 1,726,406 singleton calves born in 8,975 dairy herds between the years 2015 and 2022, inclusive; all herd-years retained had ≥50 calving events in that year. All calves were born to cows parity 15 or less whose breed composition of some combination of Holstein-Friesian, Jersey, Montbeliarde, Normande, and Norwegian Red constituted >75% of the recorded breed composition of the cow. No cow retained calved younger than 660 d of age, nor calved >2 yr from the median age at calving within parity. Dam parity number was recoded as 1, 2, 3, 4–6, and 7–15. All calves were sired by a purebred beef or dairy bull; Holstein-Friesian was considered purebred for the purpose of the present study. Ten beef breeds were represented in the data set with the major breeds being Angus (45% of data), Hereford (34% of the data), Belgian Blue (10% of the data), and Limousin (6% of the data). It is a legal requirement to record the birth-date of each calf born in Ireland as well as its dam, its sex, and its breed. It is also a legal requirement to properly dispose of and record the date of death of all bovines; this enabled the creation of a perinatal mortality trait which, in the present study, was defined as a calf dead at birth or within 24 h.

Calving assistance in Ireland is subjectively scored by producers on a 4-point scale approximating a linear increase in the duration and force required to deliver a calf: (1) no assistance required; (2) slight assistance (assistance, without the need for a mechanical aid); (3) considerable assistance (assistance aided by a mechanical device); (4) veterinary assistance (including cesareans). For the purpose of the present study, the 4-point calving assistance score was dichotomized into some assistance (i.e., scores 2, 3, and 4) or no assistance (i.e., score 1) required as well as some dystocia (i.e., scores 3 and 4) or no dystocia (i.e., scores 1 and 2) experienced at calving. Gestation length was defined as the difference, in days, between the date of conception (as dictated by the last recorded service date) and the date of calving; only gestation length records between 271 and 300 d were retained. Both dairy × dairy and beef × dairy calves were retained in the analysis of calving performance.

A proportion of calves born in dairy herds are sold at livestock auctions at a few weeks of age (Mc Hugh et al., 2010). Of the calves with a recorded calving record, those sold in single lots <42 d of age directly from the dairy farm in which they were born were retained. Both dairy × dairy and beef × dairy calves were retained; dairy × dairy female calves were not included in the analyses as their frequency was low.

Carcass-related information recorded in Ireland includes (cold) carcass weight (kg), carcass conformation, and subcutaneous carcass fat cover score. Both carcass conformation and fat score are mechanically graded on a 1- (poor conformation; little fat cover) to 15-point scale (excellent conformation; large fat cover); more details are given by Kenny et al. (2020). Price per kilogram of carcass is also recorded. Age at slaughter was also available and only animals slaughtered between 10 and 36 mo of age were retained. The slaughter traits from the calves with recorded calving performance were retained and only those slaughtered as a heifer, steer, or young bull were considered further. Only animals that resided in the farm where they were subsequently slaughtered from for ≥100 d were retained and animals with >2 inter-herd movements during their lifetime were discarded. Dairy × dairy heifers were not considered in the analysis of any slaughter trait.

Contemporary groups of herd-year-season of calving for the calving traits, and herd-year-sex-season of slaughter for the carcass traits, were defined using an algorithm routinely used in Irish national genetic evaluations (Berry et al., 2013); sex was defined as either (1) heifers, (2) steers, or (3) bulls, all of which tend to be managed differently on farm, thus requiring allocation to separate contemporary groups. The contemporary group for age at slaughter was herd-sex-season of entry onto the finishing farm where animals of different ages were grouped together (Berry et al., 2017). A maximum of 60-d duration was allowed for each contemporary group, and each contemporary group had to have ≥10 records for the calving-related traits and ≥5 for the slaughter-related traits. The contemporary group for the calf price traits was herd-date of sale; an edit of ≥5 records per contemporary group was imposed.

Following all editing, dystocia data were available on 1,389,670 births from 768,738 cows in 6,885 dairy herds; of these births, 414,642 were beef × dairy births. Of all the births, 808,767 also had a valid gestation length of which 167,548 were beef × dairy. A total of 106,623 of the calves with calving difficulty information had a recorded calf auction price, of which 53,688 were beef × dairy calves. Furthermore, postediting, of the animals with calving difficulty information, 198,607 of those who were slaughtered from 6,945 herds had carcass information, of which 91,554 were beef × dairy.

All association analyses were undertaken in ASREML (Gilmour et al., 2009) to quantify the difference in the dependent variable between, in particular, male and female calves sired by beef bulls. Both dam and contemporary group were included in all models as random effects. The binary dependent variables were all modeled using a logit link function accounting for the binominal distribution of the errors; linear models were used in the association analyses of the other continuous and normally distributed variables. All models included the fixed effects of dam parity, age at calving centered within parity (categorical effect, each 6 mo in duration ± 2 years before the median age per parity), calf sex, and whether the sire was of dairy or beef origin. Two-way interactions between calf sex and parity as well as between calf sex and whether the sire was a dairy or beef sire were also explored. Direct EBV of the calf for the dependent variable under investigation was also considered as a covariate in the models to test if it affected the difference in estimated animal sex model solutions. When the dependent variable was calving assistance or dystocia, the maternal EBV of the dam for dystocia was also considered.

The mean incidence of calving assistance, dystocia, and perinatal mortality in the edited data set was 12.00%, 1.96%, and 1.69%, respectively. The association between calf sex and calving dystocia did not differ by parity but differed by whether the sire was of beef or dairy origin. Adjusting for genetic merit for calving difficulty or even the breed composition of the dam did not influence the differential in model solutions for the sex effects. The odds of a difficult calving was greater for males, equating to an odds of 2.05 (95% CI: 1.98 to 2.13) relative to females in dairy and 2.21 (95% CI: 2.14 to 2.28) in beef. These odds translated to a predicted probability of dystocia in a beef-sired male and female calf born to a third parity cow of median age of 3.4% and 1.6%, respectively; the respective predicted probability for dairy-sired calves in the present study was 1.7% and 0.8%.

The association between the requirement for assistance at calving and calf sex also did not differ by parity but did differ by whether the sire was of a dairy or a beef breed. Based on a third-parity cow of median age at calving, the predicted probability of requiring assistance for the birth of a beef-sired male and female calf was 18.1% and 10.5%, respectively; the respective predicted probabilities for dairy-sired calves in the present study were 9.6% and 5.3%. More calving complications in male calves have already been documented in dairy herds (Mee et al., 2008; Eriksson et al., 2020) with previous literature suggesting not all is due to inter-sex differences in calving dystocia (Mee et al., 2008). What it does indicate, however, in absolute terms is that the expected calving dystocia or assistance for males is approximately twice that of females, which, given the documented association between difficult calvings and cow performance (Dematawewa and Berger, 1997; Berry et al., 2019), cannot be ignored.

Calf sex was associated with the likelihood of perinatal mortality with the odds of a beef-sired male calf dying within 24 h of birth being 1.31 times (95% CI: 1.24 to 1.38) that of a contemporary beef-sired female calf. The predicted probability of perinatal mortality in a beef-sired male and female calf born to a third-parity cow of median age was 1.5% and 1.2%, respectively; the respective predicted probability for dairy-sired calves in the present study was 1.4% and 1.2%. The predicted probability of a male (female) beef-sired calf dying within 24 h of birth was 3.8% (2.9%) in first-parity dams. Therefore, although a statistical association between calf sex and perinatal mortality existed (*P* < 0.001), no doubt assisted by the very large data set used in the preset study, the actual biological significance of the difference between both sexes was small. The association between sex and perinatal mortality did not differ by dam parity or whether or not the calf was sired by a beef or dairy sire. Furthermore, adjusting for genetic merit for perinatal mortality or breed of the dam did not influence the differential in model solutions for the sex effects, which is not unexpected because the genetic merit of a sire for perinatal mortality is unlikely to alter the sex ratio, nor is the effect of the breed of the dam. Greater perinatal mortality in male calves from dairy cows has been reported previously (Mee et al., 2008; Eriksson et al., 2020), although not always (Ring et al., 2018), and where associations have been reported, the respective relative difference between sexes has also been reported to be relatively small (Mee et al., 2008), consistent with what was observed in the present study.

The predicted marginal mean (SE) gestation length for male and female beef-sired calves was 281.7 (0.04) and 282.5 (0.04) d, equating to a 0.8 d longer gestation in males; this was less than the difference of 1.1 d when comparing the dairy-sired male (281.0 d) and female (279.9 d) calves. Including either genetic merit for gestation length or dam breed in the statistical model did not affect the relative difference between sexes in gestation length. The longer mean gestation of male versus female calves is well known in dairy cattle (Norman et al., 2009; Fouz et al., 2013), but no distinction seems to have ever been made depending on whether the calf was sired by a dairy or a beef breed. Results from the present study indicate that indeed such an interaction exists (*P* = 0.003), which, in absolute terms was small (0.3 d), but relative to the actual sex difference (0.8 to 1.1 d) was quite substantial.

Male beef-sired calves sold at auctions <42 d of age were worth, on average, €32.40 more than beef-sired female calves with the mean value of the female and male beef-sired calves being €150.7 (SE = €2.05) and €183.1 (SE = €2.04), respectively; the mean value of male dairy-sired calves was €59.44 (SE = €1.82). The residual standard deviation in the beef-sired male and female calves from the mixed model used in the analysis was €36.4 and €39.4, respectively; these values were €73.48 and €74.15 when herd-date was not included as a random effect in the model. Hence the mean difference of €32.40 between both sexes of beef-sired calves was less than the residual standard deviation in the price of beef-sired calves of the same sex sold in any given day and less than half a standard deviation when considered across time. In an analysis of animal price data from Irish dairy herds (not overlapping with the present study), Mc Hugh et al. (2010) reported a €69.47 premium for bull calves relative to heifer calves sold up to 12 wk of age; the data from Mc Hugh et al. (2010) included both dairy and beef-sired calves, although adjustments were made for sire breed (as well as other factors) in their statistical model. Discrepancies in the actual difference in price between studies not overlapping in time is expected because of the volatility in markets; the association between calf sex in beef-sired calves in the present study differed by year, albeit the mean sex difference only varied from €21.40 to €45.45 across years.

The predicted marginal means for the slaughter traits for beef-sired heifers, steers, and bulls are in Table 1. Adjusting for either genetic merit or the breed composition of the dam did not affect the relative difference in performance between sexes. Several different production systems are advocated for beef × dairy heifers, steers, and bulls in Ireland (Supplemental Table S1; https://figshare.com/articles/figure/Supplementary_Table_1_docx/13697386). Both heifers and steers in Ireland are generally farmed extensively, exploiting the cost competitiveness of grazed pasture linked to the comparative advantage of Ireland's ability to grow large quantities of high-quality pasture in a climate that is also very conducive to grazing the pasture in situ for a large portion of the year. Bulls, in contrast, are generally intensively fed after an initial summer grazing (Supplemental Table S1). Relative to heifers, the carcasses of steers and bulls were 56.9 and 55.4 kg heavier, respectively. In an analysis of >1.1 million carcass records from various breed crosses on Swedish dairy cows, Eriksson et al. (2020) reported the carcasses of bulls and steers to be between 30 and 51 kg heavier than heifers, representing 11% to 18% of the mean carcass weight of the heifers. The carcasses of bulls were, on average, 1.09 units superior (*P* < 0.001) in conformation score to those of heifers in the present study, who, in turn, were slightly (0.06 units) superior to those of steers; these mean results compare well with those of Eriksson et al. (2020), who also reported mean conformation scores (same system of carcass conformation scoring as used in the present study) of bulls to be 0.4 to 0.9 units higher than heifers depending on the breed crosses, whereas steers were up to 0.4 units inferior to heifers. Heifers in the present study were slaughtered, on average, 79.1 d younger than steers, although heifers were slaughtered, on average, 93.8 d older than bulls. Nonetheless, heifers were fatter than both steers and bulls so could actually have been slaughtered younger (albeit then probably with lighter carcasses). Age was not considered as a covariate in the statistical model for the carcass traits and nor was carcass weight included in the statistical model for age; this was undertaken so as to not necessarily detect a sex effect per se but instead a system effect, which, in Ireland at least, is synonymous with animal sex. Adjusting, for example, heifer carcass weights for inter-sex differences in age at slaughter would not reflect reality because the heifer carcasses would, on average, likely be overfat for slaughter. While price per kilogram was not hugely different between sexes, when sex differences in carcass weight were considered, the mean value of heifers, steers, and bulls was €1,089, €1,296, and €1,233, respectively; the mean difference in carcass value between males and females of €175.68 is larger than the respective difference of €32.40 as calves, but this is expected given it would have to include a profit margin for the producer purchasing the calf.

**Table 1:** Number of records (N) per sex as well as the respective marginal mean (SE in parentheses) for carcass weight, conformation,^1^ fat,^1^ price per kilogram, and age at slaughter

Trait	Heifers	Steers	Bulls
N	39,720	45,011	6,823
Weight (kg)	280.0 (0.68)	336.9 (0.6)	335.4 (0.86)
Conformation (units)	5.09 (0.02)	5.03 (0.02)	6.18 (0.03)
Fat (units)	9.26 (0.03)	8.40 (0.03)	6.5 (0.04)
Price per kg (€ cents)	388.8 (0.47)	384.6 (0.37)	367.6 (0.60)
Age (d)	708.8 (1.57)	787.9 (1.57)	616.6 (1.90)

1 Scored on a scale from 1 (lowest) to 15 (highest) in accordance with the EUROP (E, U, R, O, P = excellent, very good, good, fair, poor) grading system.

In nonexpanding dairy herds with good reproductive performance, the use of sex-sorted semen has facilitated a greater use of beef-on-dairy to potentially increase the value of the resulting calf (Mc Hugh et al., 2010), thus adding to farm revenue. Using the approach for calculating economic values for a beef-on-dairy breeding index (Berry et al., 2019) along with an economic value calculated since for age at slaughter, it was possible to calculate the mean expected difference in profit between beef-sired females and males (steers were considered to represent males) based on the calculated mean difference in performance in the present study for some of the traits in the breeding index (Table 2). While the benefit of fewer complications at calving and shorter gestation favored heifers, this was more than offset by their lighter carcasses, with the younger age at slaughter also favoring the heifers (Table 2). In total, the mean expected difference in profit between both sexes was just €1.36 in favor of males. However, given their lighter live weights and earlier slaughter, the carbon footprint of heifers may be lower.

**Table 2:** Expected average difference in profit between steers and heifers based on calculated sex differences in mean performance in the present study as well as the associated economic values per trait

Trait	Economic value (€)	Heifer – steer	Monetary value (€)
Calving^1^			
Primiparous		4.0% dystocia + 7.9% assistance^2^	28.39
Multiparous		1.8% dystocia + 5.8% assistance^2^	13.35
Stillbirth, units	−172	−0.004	0.69
Gestation, d	−7.47	−0.8	5.98
Carcass weight, kg	2.29	−56.9	−130.30
Carcass conformation^3^	10.92	0.059	0.64
Age at slaughter, d	−1.01	−79.1	79.89
Total			−1.36

1 No economic value provided, as the monetary cost was directly calculated from an underlying liability scale and the difference in sex thresholds imposed on the distribution as per Berry et al. (2019).

2 Assistance incidence is based on the predicted probability of assistance calculated for each sex by parity minus the respective predicted probability of dystocia (to enable separate monetary values to be allocated to each).

3 1 (poor) to 15 (excellent).

The most important traits for the majority of dairy producers when generating surplus calves are gestation length and calving difficulty owing to their contribution to herd profit (Dematawewa and Berger, 1997; Berry et al., 2019). Although large intra-breed variability in cattle performance is known to exist (Berry and Ring, 2020a; Eriksson et al., 2020), many (but not all) modern beef breeds mated to dairy cows have, on average, longer gestations than their dairy counterparts (Fouz et al., 2013; Berry and Ring, 2020a) and are more genetically predisposed to complications at calving (Fouz et al., 2013; Berry and Ring, 2020a; Eriksson et al., 2020). Therefore, dairy producers tend to specifically choose beef sires or breeds with expected short gestation progeny that will be born with minimal intervention. No inferred genetic correlation exists between gestation length and either carcass weight (−0.06) or conformation (−0.04) in Angus (Berry et al., 2019), but heavier, more-conformed carcasses are genetically associated with a greater expected calving difficulty in beef (Berry et al., 2019); the partial correlations (adjusted for sire breed) between direct calving difficulty and both carcass weight and conformation in the present study based on the 6,862 beef sires used were 0.41 and 0.26, respectively. Although breeding indexes now exist to concurrently select for improved calving and carcass performance (Berry et al., 2019), results from the present study suggest that only using X-chromosome bearing beef sperm could also contribute to shorter gestation length and easier calving with no compromise in carcass conformation relative to steers; the carcass weight of the heifers was lighter than that of the male cattle, albeit they were slaughtered 79 d younger than their steer counterparts.

The regression coefficient of the logit of phenotypic dystocia score on the direct EBV for calving difficulty in the present study was 0.1637. Therefore, the mean difference in predicted probability of calving dystocia between males and female beef-sired calves of 1.8 percentage units equates to a difference in direct EBV of the calf of 4.98 percentage units. Based on a within-breed regression coefficient of 3.15 when regressing direct calving difficulty EBV on the EBV for carcass weight, this implies that by using heifers and relaxing the genetic merit for calving difficulty to result in phenotypic calving difficulty the same as what was achieved for male calves in the present study, the EBV of the calves for carcass weight could be, on average, 15.7 kg heavier; sires with superior genetic merit for conformation (with an associated expected mean increase in calving difficulty) could possibly also be facilitated. Hence, using X-chromosome sorted semen could enable sires with superior genetic merit for carcass weight or conformation to be used.

In conclusion, many benefits exist for beef-sired heifer calves in that they have, on average, shorter gestations with less expected assistance required at calving, and although their calf value is less and their carcasses lighter than their male counterparts, they are slaughtered several months younger with no compromise in carcass conformation relative to steers. In monetary terms, little difference existed between males and females, although the difference will largely be dictated by the ratio of milk price (i.e., the impact of calving difficulty) to meat price (i.e., the impact on carcass traits) and feed costs (i.e., age at slaughter).

## References

[bib1] Berry D.P. (2021). Invited review: Beef-on-dairy—The generation of crossbred beef × dairy cattle. J. Dairy Sci..

[bib2] Berry D.P., Amer P.R., Evans R.D., Byrne T., Cromie A.R., Hely F. (2019). A breeding index to rank beef bulls for use on dairy females to maximise profit. J. Dairy Sci..

[bib3] Berry D.P., Cromie A.R., Judge M.M. (2017). Large exploitable genetic variability exists to shorten age at slaughter in cattle. J. Anim. Sci..

[bib4] Berry D.P., Kearney J.F., Twomey K., Evans R.D. (2013). Genetics of reproductive performance in seasonal calving dairy cattle production systems. Ir. J. Agric. Food Res..

[bib5] Berry D.P., Ring S.C. (2020). Observed progeny performance validates the benefit of mating genetically elite beef sires to dairy females. J. Dairy Sci..

[bib6] Berry D.P., Ring S.C. (2020). Short communication: The beef merit of the sire mated to a dairy female impacts her subsequent performance. J. Dairy Sci..

[bib7] Dematawewa C.M.B., Berger P.J. (1997). Effect of dystocia on yield, fertility, and cow losses and an economic evaluation of dystocia scores for Holsteins. J. Dairy Sci..

[bib8] Eriksson S., Ask-Gullstrand P., Fikse W.F., Jonsson E., Eriksson J.-Å., Stålhammar H., Wallenbeck A., Hessle A. (2020). Different beef breed sires used for crossbreeding with Swedish dairy cows—Effects on calving performance and carcass traits. Livest. Sci..

[bib9] Fouz R., Gandoy F., Sanjuán M.L., Yus E., Diéguez F.J. (2013). The use of crossbreeding with beef bulls in dairy herds: Effects on calving difficulty and gestation length. Animal.

[bib10] Gilmour A.R., Cullis B.R., Welham S.J., Thompson R. (2009).

[bib11] Johanson J.M., Berger P.J. (2003). Birth weight as a predictor of calving ease and perinatal mortality in Holstein cattle. J. Dairy Sci..

[bib12] Kenny D., Murphy C.P., Sleator R.D., Judge M.M., Evans R.D., Berry D.P. (2020). Animal-level factors associated with the achievement of desirable specifications in Irish beef carcasses graded using the EUROP classification system. J. Anim. Sci..

[bib13] Mc Hugh N., Fahey A.G., Evans R.D., Berry D.P. (2010). Factors associated with selling price of cattle at livestock marts. Animal.

[bib14] Mee J.F., Berry D.P., Cromie A.R. (2008). Prevalence of, and risk factors associated with, perinatal calf mortality in pasture-based Holstein-Friesian cows. Animal.

[bib15] Mee J.F., Berry D.P., Cromie A.R. (2011). Risk factors for calving assistance and dystocia in pasture-based Holstein-Friesian heifers and cows in Ireland. Vet. J..

[bib16] Norman H.D., Wright J.R., Kuhn M.T., Hubbard S.M., Cole J.B., VanRaden P.M. (2009). Genetic and environmental factors that affect gestation length in dairy cattle. J. Dairy Sci..

[bib17] Ring S.C., McCarthy J., Kelleher M.M., Doherty M.L., Berry D.P. (2018). Risk factors associated with animal mortality in pasture-based, seasonal-calving dairy and beef herds. J. Anim. Sci..

[bib18] Van Doormaal B. (2019). Use of beef sire semen in the dairy industry. https://www.cdn.ca/document.php?id=526.

